# P0.1 is an Unreliable Measure of Effort in Support Mechanical Ventilation in Comparison With Esophageal-Derived Measures of Effort: A Comparison Study

**DOI:** 10.1097/CCM.0000000000006745

**Published:** 2025-06-11

**Authors:** Florence E. Smits, Petra J. Rietveld, Jacob W.M. Snoep, Franciska van der Velde-Quist, Evert de Jonge, Abraham Schoe

**Affiliations:** 1 All authors: Department of Intensive Care, Leiden University Medical Center, Leiden, The Netherlands.

**Keywords:** esophageal pressure swings, intensive care unit, mechanical ventilation, P0.1, pressure-time product, work of breathing

## Abstract

**OBJECTIVE:**

Respiratory effort should be assessed in patients on mechanical ventilation in support mode. With the insertion of esophageal balloons, it is possible to measure different parameters of effort, such as change in esophageal pressure (ΔPes), work of breathing (WOB), and pressure-time product (PTP). Although some studies advocate for the use of P0.1 as a measure of effort, there is significant controversy as it is considered more a measure of respiratory drive. This study investigates the correlation between P0.1 and esophageal-derived parameters of effort.

**DESIGN:**

This was a retrospective observational comparison study.

**SETTING:**

This study is conducted in the mixed medical-surgical ICU at the Leiden University Medical Center (Leiden, The Netherlands).

**PATIENTS:**

Data were collected from 30 mechanically ventilated patients in spontaneous breathing mode.

**INTERVENTIONS:**

None.

**MEASUREMENTS AND MAIN RESULTS:**

From each patient, a minimum of three different time frames of 5 minutes were used to collect the average values of P0.1, WOB, PTP, and ΔPes over this time frame. Statistical models accounting for repeated measurements were applied to assess correlations among these parameters. In total, 117 timeframes were analyzed from 39 patient cases. The analysis revealed poor correlations between P0.1, as measured in this study, and WOB (*R*^2^ = 0.111), PTP (*R*^2^ = 0.113), and ΔPes (*R*^2^ = 0.034), whereas the esophageal-derived parameters showed high correlations with each other (PTP vs. WOB, *R*^2^ = 0.886; ΔPes vs. WOB, *R*^2^ = 0.848; and ΔPes vs. PTP, *R*^2^ = 0.876).

**CONCLUSIONS:**

The results demonstrated poor correlations between P0.1 and the other effort parameters, whereas strong correlations were observed among ΔPes, WOB, and PTP. These findings underscore the need for careful consideration of monitoring tools to ensure appropriate assessment and management, and the importance of using esophageal catheters for accurate monitoring of respiratory effort, particularly in spontaneously breathing patients.

**KEY POINTS**

**Question:** Does P0.1 correlate with esophageal-derived measures of respiratory effort in mechanically ventilated patients?

**Findings:** In this observational study analyzing 117 timeframes from 30 patients, P0.1 showed poor correlations with work of breathing, pressure-time product, and esophageal pressure swings, whereas strong correlations were observed between the esophageal-derived parameters.

**Meaning:** Accurate assessment of respiratory effort in mechanically ventilated patients requires esophageal catheters, as P0.1 alone may not provide reliable measurements.

When a patient is on mechanical ventilation in support mode, it is important to monitor their respiratory effort, as excessive effort in a vulnerable lung can lead to adverse outcomes (1). Premature attempts at spontaneous effort during mechanical ventilation in injured lungs can result in complications such as ventilator-induced lung injury (VILI) or patient-self-inflicted lung injury, influenced by the lung’s stress-strain dynamics (2–4). High patient effort is widely considered the primary cause of these adverse effects, making accurate estimation of patient effort essential (1, 5, 6).

Respiratory drive refers to the intensity of the neural stimulus from the brain stem to the respiratory muscles (7). In contrast, respiratory effort is the physical work performed by the respiratory muscles to achieve ventilation (8). Although respiratory drive can be assessed using noninvasive methods like P0.1, measuring respiratory effort often requires more invasive techniques such as esophageal pressure (Pes) monitoring.

To monitor respiratory effort, parameters such as work of breathing (WOB), pressure-time product (PTP), and esophageal pressure swings (ΔPes, change in esophageal pressure) can be used. However, all these respiratory effort parameters require the insertion of an esophageal balloon (1, 6, 9–11). WOB, measured in joules per minute (J/min), reflects the energy needed for spontaneous ventilation by integrating volume over Pes (12–15). PTP, measured in cm H_2_O·s/min, takes the isometric phase of muscle contraction into account and is particularly useful in patients with weak respiratory efforts, where WOB may not be accurately measured (12, 15, 16). PTP is calculated as the time integral of the Pes (15). ΔPes is a surrogate for muscular pressure (Pmus), generated by the diaphragm, following the equation Pmus = Pcw (chest wall pressure) – Pes (1, 9, 17). Pcw can be calculated as the product of tidal volume and chest wall elastance (Ecw), but is often neglected in daily practice because Ecw is often relatively low (17). ΔPes is calculated as the difference between minimum and maximum Pes values per breath, making it relatively easy to obtain this parameter.

P0.1 reflects the respiratory drive by measuring the negative pressure at the airway opening 0.1 seconds after the start of an inspiratory effort during the occlusion of the inspiratory valve. It is a noninvasive method, recognized for its simplicity (1, 11, 18–21). The theory behind P0.1 is that there is no neurologic feedback to the brain stem that provides information about the compliance of the system, which increases the neurologic output from the brain stem to the diaphragm and other respiratory muscles, thereby increasing effort when compliance is low. P0.1 is designed to measure the neurologic or respiratory drive of the patient, not the effort the patient is putting in a breath (22).

In certain ventilators, P0.1 is derived using a quasiocclusion method, allowing it to be displayed breath-by-breath on the ventilator or in a patient data management system (23). This facilitates monitoring of P0.1 trends. Although it is primarily used for estimating respiratory drive, P0.1 is also advocated in some studies as a measure of effort (1, 18, 24–27). P0.1 is easier to obtain compared with WOB, PTP, and ΔPes, all requiring the insertion of an esophageal catheter.

Despite the advantages of P0.1, the validity and reliability as a measure of respiratory effort are controversial. Proponents argue that P0.1 is a valuable, noninvasive metric that accurately reflects the effort of breathing, being unaffected by the compliance and resistance of the respiratory system, thus directly reflecting the respiratory center’s output (1, 8, 18, 24–27). Critics, however, highlight several limitations and potential sources of error, noting that P0.1 measures drive rather than effort and depends on multiple variables that can affect its accuracy (11, 18–21). Furthermore, there is an ongoing debate about the accuracy of quasiocclusion-derived P0.1 values in estimating respiratory effort (28).

Given these discrepancies, the aim of this study was to investigate the correlation between established methods for measuring patient effort (WOB, PTP, and ΔPes) and the P0.1 value obtained via the quasiocclusion method.

## MATERIALS AND METHODS

### Study Design

This study used data from two ongoing single-center observational studies, between December 2022 and April 2024, both approved by the hospital investigational review board (METC Leiden, Delft, Den Haag). The study titled “Oxygen Consumption (VO_2_), Effort, and Weaning in the Mechanically Ventilated Patient in the Intensive Care Unit (ICU) (EXTUBATE)” was approved on November 10, 2023, under protocol ID P23.068. The study titled “Automated Detection of Patient Ventilator Asynchrony Using Pes Signal” was approved on December 9, 2022, under protocol ID 2022-061. These studies are registered at ClinicalTrials.gov under the identifiers NCT06391424 and NCT06186557, respectively. Written informed consent was obtained from the patient’s next-of-kin. All procedures were conducted in accordance with the ethical standards of the responsible committee on human experimentation and with the Declaration of Helsinki (1975) and its later amendments.

### Patients

Patients were recruited from the mixed medical-surgical ICU of the Leiden University Medical Center (LUMC, Leiden, The Netherlands). Eligible patients had acute respiratory failure and were receiving spontaneous mechanical ventilation. Additional inclusion criteria were patients 18 years old or older with an esophageal balloon catheter in situ. Both Cooper Surgical (Cooper Surgical, Trumbull, CT) and NutriVent (Sidam, Mirandola, Italy) esophageal catheters were used to measure Pes. All patients were ventilated with a Hamilton C6 mechanical ventilator (Hamilton-AG, Bonaduz, Switzerland). There were no specific exclusion criteria.

### Data Collection

Demographic data, including age, sex, body mass index, Acute Physiology and Chronic Health Evaluation-IV scores, ICU length of stay, and total duration of mechanical ventilation, were collected for all included patients. Waveform data for Pes, airway pressure (Paw), and P0.1 were gathered from the ventilators at a sampling rate of 50 Hz. Pes measurements were used to calculate the WOB, PTP, and ΔPes (9, 12–17). P0.1 values were obtained from the Hamilton C6, which employs the aforementioned quasiocclusion method to measure P0.1 in spontaneously breathing patients (29). During the initiation of a breath, a negative deflection in the Paw signal occurs before the ventilator provides positive pressure support. This negative deflection, typically shorter than 0.1 seconds, is extrapolated to a full 0.1-second measurement, yielding the P0.1 value. Details of the quasiocclusion method used by the Hamilton C6 are provided in **Supplementary 1** (https://links.lww.com/CCM/H744) (29).

Before conducting measurements, the esophageal balloon was calibrated according to the standard LUMC calibration protocol to ensure accurate values (30). A ΔPes to ΔPaw ratio between 0.8 and 1.2 during an occlusion was considered reliable (9, 31).

### Signal Analysis

Three random time frames of five consecutive minutes were selected per patient case, to obtain the means for each parameter of effort. As some patients were measured at multiple points during their ICU admission, they contributed to more cases. Each measurement yielded three randomly selected 5-minute time frames.

Each 5-minute frame was inspected to ensure reliable measurements. If a 5-minute frame contained an error leading to erroneous values, an alternative 5-minute time frame was selected.

All preprocessing signal analysis steps were performed using Python (Spyder IDE 5.4.3, Python 3.11; Python Software Foundation, Beaverton, OR).

### Statistical Analysis

Descriptive statistics were reported in the appropriate format based on the data characteristics. Continuous variables were summarized as mean (± sd) or median (interquartile range), whereas categoric variables were presented as count (percentage).

For inferential statistics, normality was assessed using histograms, Q-Q plots, and the Shapiro-Wilk test. Log-transformed data were used if the data were not normally distributed. If the distribution remained nonnormal after log transformation, nonparametric statistics were employed.

To analyze the relationships between the different parameters while accounting for repeated measurements, as there were three-time frames for each patient case, and some patients were measured in both databases, we employed both generalized estimating equations (GEE) and generalized linear mixed-effects models (GLMM). Each method served a specific purpose.

GEEs were used to estimate population-averaged effects. The models were fit using the geeglm function in R (R Foundation for Statistical Computing, Vienna, Austria) (32). To determine the most appropriate correlation structure of the model, we compared the quasilikelihood under the independence model criterion (QIC) values and selected the model with the lowest QIC. Each GEE model included the predictor variable of interest and controlled for the repeated measures within subjects.

GLMMs were used to explore the subject-specific variability, random effects, whereas also providing a measure of correlation, *R*^2^ values. Models were performed on standardized data to account for measurements in different units across parameters. Standardization between units was established using the mean and sd of each variable. Each GLMM included a random intercept to account for variability between subjects and fixed effects for the intercept and predictor variable of interest. The models allowed us to compute marginal *R*² values, which were used to assess the correlations.

To interpret the strength of relationships, correlations were categorized as follows: less than 0.30, poor/negligible; 0.31–0.50, low; 0.51–0.7, moderate; 0.71–0.9, high; greater than 0.91 very high (33).

In total, six models (GEEs and GLMMs) were created to compare all parameters with each other.

Statistical significance was defined as a *p* value of less than 0.05. All statistical analyses were performed using RStudio, version 4.3.1 (R Core Team 2023, Vienna, Austria).

## RESULTS

A total of 30 patients (20 male [67%]), with a mean age of 60.8 (±10.5) years provided 39 cases for analysis between December 2022 and April 2024. In total, 117 time frames were analyzed. A demographic overview is provided in **Table 1**. The median ICU length of stay was 19.5 (15–48.25) days, and the median ventilation duration was 17.3 (11.1–39.2) days.

**TABLE 1.** Baseline Patient Characteristics

Clinical Characteristic	Overall (*n* = 30)
Gender, male, *n* (%)	20 (66.7)
Age, mean (sd)	60.8 (10.5)
Body mass index, mean (sd)	28.8 (6.1)
Acute Physiology and Chronic Health Evaluation-IV scores, median (IQR)	69.0 (52.5–100.5)
Days of ICU length of stay, median (IQR)	19.5 (15–48.25)
Days of mechanical ventilation, median (IQR)	17.3 (11.1–39.2)

IQR = interquartile range.

Descriptive statistics for clinical characteristics are shown with continuous variables presented as median with interquartile range (25th–75th percentiles) or mean with sd, depending on their contribution, and categoric variables presented as counts with percentage (*n* [%]).

The Shapiro-Wilk test indicated that the data were not normally distributed, even after log-transformation. Median values of the effort parameters are summarized in **Table 2**. All values were within the expected normal ranges for mechanically ventilated patients (6, 34, 35).

**TABLE 2.** Median Values and Interquartile Ranges Per Parameter

Parameter	Median Value	IQR
P0.1	4.73 cm H_2_O	1.97–6.77 cm H_2_O
Change in esophageal pressure	6.23 cm H_2_O	3.94–9.21 cm H_2_O
Work of breathing	4.53 J/min	2.60–6.78 J/min
Pressure-time product	75.11 cm H_2_O·s/min	46.83–109.63 cm H_2_O·s/min

IQR = interquartile range.

The measured parameters are represented as their measured median value and interquartile range (25th–75th percentile). These are the median values of all 117 timeframes used in the analysis.

**Figure 1.**
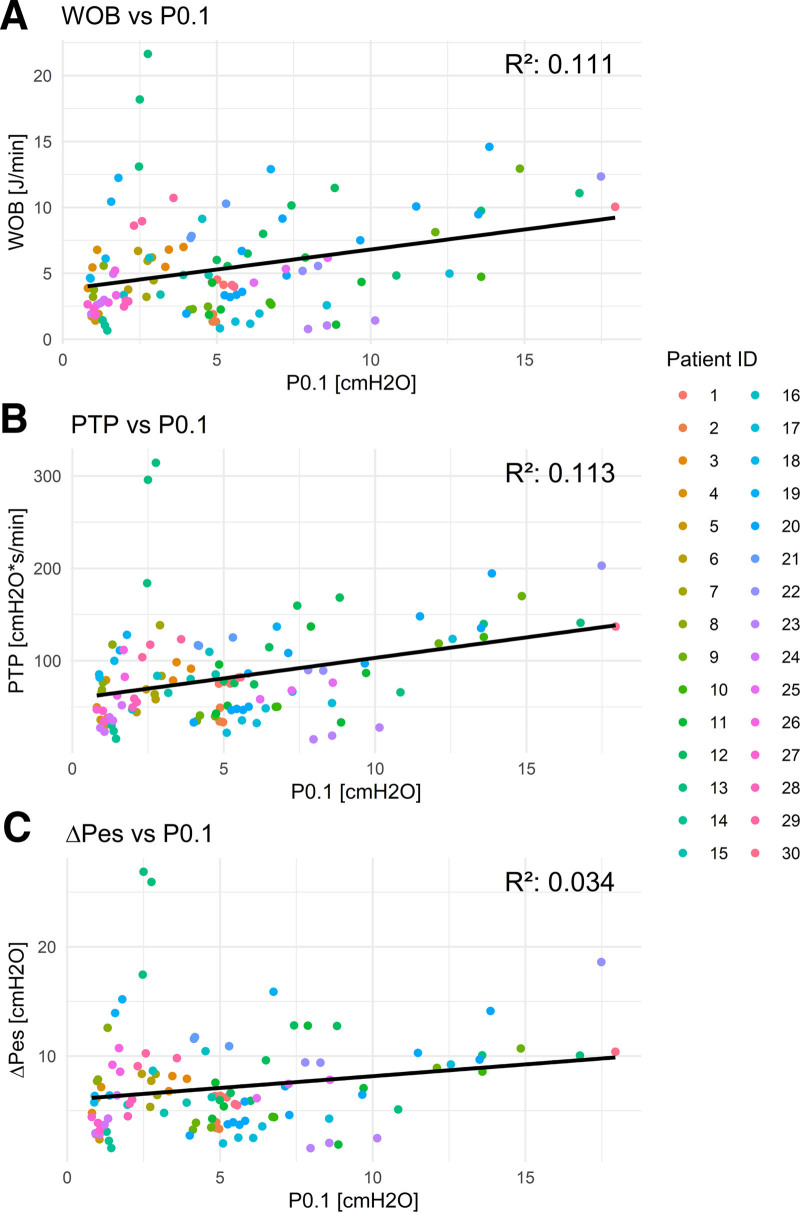
Generalized estimating equations regression line of the esophageal-derived parameters in comparison with the P0.1 with the *R*^2^ value derived from the generalized linear mixed-effects models marginal *R*^2^. Work of breathing (WOB) compared with P0.1 (**A**), pressure-time product (PTP) compared with P0.1 (**B**), and esophageal pressure swing (ΔPes) compared with P0.1 (**C**), all showed poor correlations.

**Figure 2.**
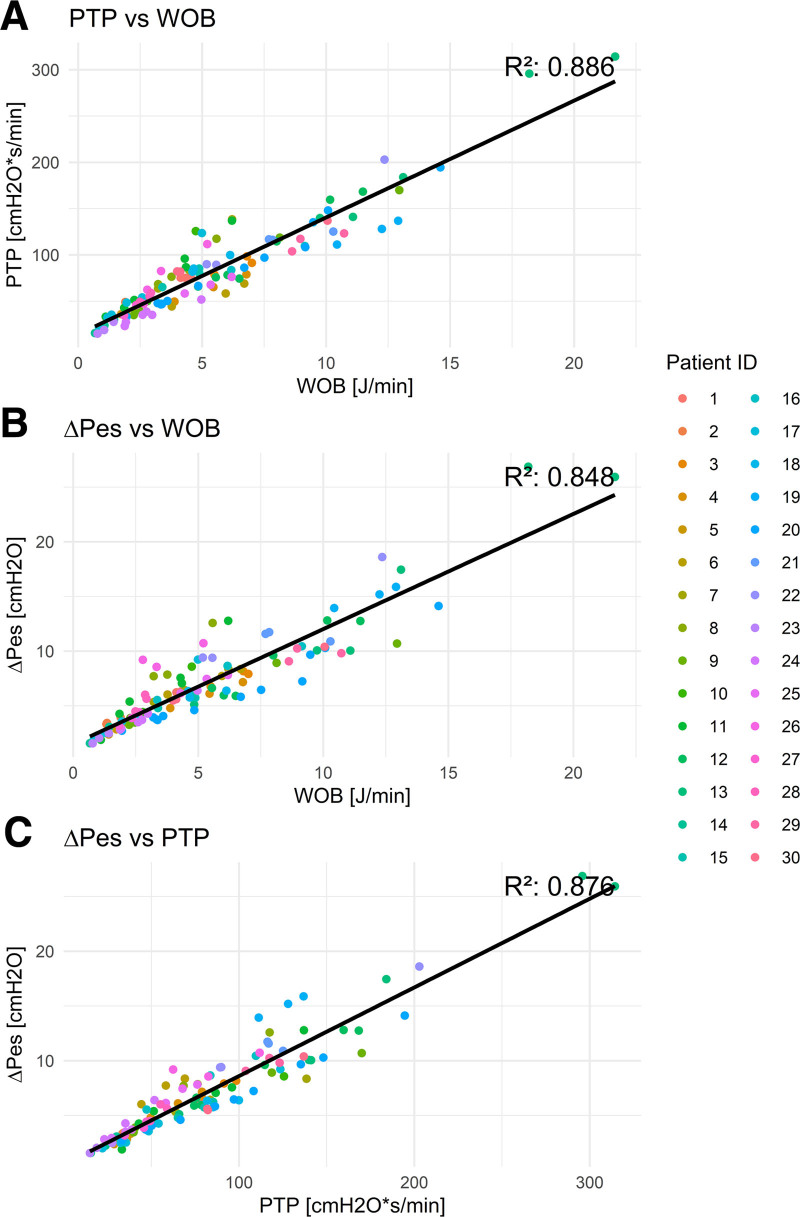
Generalized estimating equations regression line of the esophageal-derived parameters in comparison with each other with the *R*^2^ value derived from the generalized linear mixed-effects models marginal *R*^2^. Work of breathing (WOB) compared with pressure-time product (PTP) (**A**), WOB compared with esophageal pressure swing (ΔPes) (**B**), and PTP compared with ΔPes (**C**), all showed high correlations.

The marginal and conditional *R*^2^ values of the various GLMM models are described in **Table 3**. Models incorporating esophageal-derived effort parameters demonstrated superior correlation compared with those with P0.1 as predictors. Specifically, P0.1-related models consistently showed poor correlation, whereas models based on esophageal effort parameters had high marginal *R*^2^ values, indicating strong predictive relationships. The conditional *R*^2^ values showed that also the models of the esophageal-derived effort parameters better captured the variance of the fixed and random effects. The GEE models provided additional insights into the relationships between the physiologic parameters. The regression lines were plotted to visualize these relationships while taking the individual patients into consideration (**Figs. 1** and **2**).

**TABLE 3.** Generalized Linear Mixed Models Outcomes of Correlation With Both Conditional and Marginal *R*^2^ for Each Predictor and Their Dependent Variable

Dependent Variable	Predictor	Conditional *R*^2^	Marginal *R*^2^
WOB	P0.1	0.587	0.111
PTP	P0.1	0.444	0.113
ΔPes	P0.1	0.480	0.034
PTP	WOB	0.962	0.886
ΔPes	WOB	0.922	0.848
ΔPes	PTP	0.964	0.876

ΔPes = change in esophageal pressure, PTP = pressure-time product, WOB = work of breathing.

Marginal *R*² represents the proportion of variance explained by fixed effects alone, offering insight into the contribution of the predictors to the outcome. Conditional *R*², on the other hand, accounts for both fixed and random effects, reflecting the total variance explained by the model. Showing both metrics ensures a clear distinction between the influence of predictors and the overall explanatory power of the model, which is critical for interpreting mixed-effects models in the context of hierarchic or grouped data.

## DISCUSSION

The primary finding of this study is that P0.1, as measured in this study, has a poor correlation with ΔPes, WOB, and PTP, indicating that P0.1 is not an ideal surrogate for measuring respiratory effort in clinical practice. In contrast, ΔPes, WOB, and PTP showed strong correlations with each other, as expected, supporting their reliability as measures of respiratory effort. Although they all have the same measurement as a base, the Pes curve, these variables are not completely similar. WOB expresses the energy the patient puts into displacing volume during inhalation, whereas PTP expresses the effort needed in conditions where the resistance is high, and in isovolumetric conditions. Interestingly, we found that the ΔPes, which is a simpler parameter to obtain, was colinear as well.

Analysis of the *R*^2^ values revealed that all involving P0.1 as a predictor of any of the other effort parameters were poor. In comparison, the relationships between the different effort parameters derived from the Pes showed relatively high correlations. In the GEE models, the results with P0.1 as a predictor contain more scatter around the regression lines, again reflecting the poor predictive value of this parameter.

This discrepancy between the correlations of the measured P0.1 and esophageal-derived effort can be explained by the different methodologies of parameter measurement: Pes is a direct measure of the pressure, whereas P0.1 is extrapolated based on a part of the Paw. Esophageal measurements are closely related to physiologic parameters of respiratory effort, whereas P0.1 reflects the initial response of the respiratory system to a respiratory stimulus, influenced by both neural respiratory drive and respiratory mechanics (35). This introduces variability that is absent in Pes measurements.

Literature presents mixed findings about the use of the P0.1 as a measure of respiratory effort. For instance, de Vries et al (27) reported a good correlation between P0.1 and effort in their abstract. However, when looking at their results, the *R*^2^ value between P0.1 and lung effort was more similar to our findings, showing moderate correlations (*R*^2^ = 0.22). However, these correlations in their study were categorized differently from other thresholds, which may distort the comparison. Additionally, a recent study found no clear consensus on using P0.1 to differentiate between high and low effort (26). Furthermore, Telias et al (25) found that P0.1 only correlates moderately with diaphragm electric activity. Therefore, P0.1 is not recommended as a reliable measure of effort. WOB and PTP are increasingly investigated for integration into routine ICU decision-making (11, 19, 36, 37). Esophageal catheters, although minimally invasive, provide reliable measurements of ΔPes, WOB, and PTP (9, 12, 15).

Based on our results, we advocate the use of esophageal catheters to achieve accurate and effective monitoring of a patient’s respiratory effort. The findings of this study indicate that respiratory effort can be measured using only the ΔPes, which is relatively straightforward to assess compared with WOB and PTP when using an esophageal catheter. This method does not require any additional calculations that are difficult to perform bedside. Despite the initial promise of using P0.1 in the absence of an esophageal balloon catheter, our findings indicate that direct measurements provide a more reliable and consistent correlation with effort parameters. This suggests that patients expected to require prolonged mechanical ventilation may benefit from direct monitoring. Accurate tracking of respiratory effort with esophageal catheters could potentially reduce ICU-acquired weakness and weaning failure by enabling more precise respiratory management, allowing patients to use their muscles without overexertion. However, it is important to remain cautious as the direct impact on clinical outcomes, such as ICU-acquired weakness and weaning failure, has yet to be conclusively demonstrated. The variability introduced by indirect measures such as P0.1 underscores the need for caution when the use of surrogate markers is considered for clinical assessments.

This study has several strengths and weaknesses. The primary strength is the inclusion of data from patients admitted to a mixed medical-surgical ICU without specific restrictions on the patient selected. This approach enhances the generalizability of the results to all ICU patients spontaneously breathing on mechanical ventilation. Furthermore, this research focused on the examination of the correlation between measurements rather than the distinguishment between low and high effort in this patient group. The analysis employed both marginal and conditional *R*^2^ values, providing a comprehensive understanding of the relationships between variables by differentiating between variance explained by fixed and random effects.

However, there were some limitations. This study compared the WOB, PTP, and ΔPes with P0.1 values derived from the Hamilton C6 ventilator. Yet, several studies have examined the validity and reliability of this value and found that the quasiocclusion-derived P0.1 value differs from a directly measured P0.1 (23, 28). Takane et al (23), however, did not compare the quasistatic P0.1 with the occlusion P0.1, but with the Pes0.1 value, since it was impossible to measure airway P0.1 with occlusion on the Hamilton C6. Consequently, they used esophageal P0.1 and esophageal swing as surrogates, which may have led to different outcomes. In another study by Katayama et al (28), they concluded that the P0.1 values derived with the Hamilton C6 were less reliable than directly measured P0.1. We hypothesize that this may result from the placement of the flow sensor within the respiratory circuit and the ventilator’s use of bias flow, as it possibly results in a higher baseline and a potential underestimation of P0.1 values.

Furthermore, Pes measurements depend on balloon calibration, and an alternative optimal filling volume might have improved accuracy (30, 38). Balloon positions, however, were calibrated to derive a ratio between 0.8 and 1.2, in accordance with the standard protocol at the LUMC, with a fixed filling volume (1 cm H_2_O for the CooperSurgical and 3 cm H_2_O for the NutriVent). Mojoli et al (30) demonstrated that the optimal filling volume (Vbest) corresponded with a ratio of 1 between ΔPes/ΔPaw. Despite these limitations, we believe the results are still applicable to our ICU patients mechanically ventilated with a Hamilton C6.

Future research should compare the quasistatic P0.1 values with the occlusion pressure obtained during an expiratory hold, taking into account the potential for greater patient effort during this breath and accurate conversion of this effort to validate the comparison with P0.1 from the ventilator (27).

Additionally, the use of esophageal balloons to measure patient effort requires further investigation to assess the influence of varying filling volumes on measurement accuracy and to explore the impact of deviations from the optimal filling volume (Vbest) on the parameters. Although our findings suggest potential benefits, it is important to remain cautious as the direct impact on clinical outcomes of VILI and ICU-acquired weakness has yet to be conclusively demonstrated. The optimal use of these parameters in monitoring mechanically ventilated patients and their role in preventing VILI and ICU-acquired weakness should be assessed in daily clinical practice. As explained by van Oosten et al (8) there are a lot of different parameters to monitor effort, with Pes remaining the reference standard.

Exploring the value of the Pes-derived effort measurements could improve patient care for those undergoing mechanical ventilation. Given the reliability demonstrated in this study, esophageal catheters offer a promising approach to optimizing the management of respiratory effort in ICU patients.

## CONCLUSIONS

This study aimed to investigate the correlation between P0.1, measured with the Hamilton C6, and various measures of respiratory effort, including ΔPes, WOB, and PTP, in spontaneously breathing mechanically ventilated ICU patients, while accounting for repeated measurements. The results demonstrated poor correlations between P0.1 and the Pes-derived effort measurements, whereas strong correlations were observed among ΔPes, WOB, and PTP. These findings underscore the importance of being cautious when using P0.1 as a surrogate of respiratory effort.

## ACKNOWLEDGMENTS

We thank Nan van Geloven, a statistician at the Leiden University Medical Center, for her invaluable assistance with the statistical analysis for this study. Her help in addressing the complexities of repeated measurements significantly contributed to the rigor and clarity of our results.
